# Signal cancellation and contrast invariance in electrosensory systems

**DOI:** 10.1186/1471-2202-13-S1-F2

**Published:** 2012-07-16

**Authors:** Jorge F Mejias, Gary Marsat, Kieran Bol, Erik Harvey-Girard, Leonard Maler, Andre Longtin

**Affiliations:** 1 Department of Physics, University of Ottawa, Ottawa, K1N 6N5 Ontario, Canada; 2 Department of Cellular and Molecular Medicine, University of Ottawa, Ottawa, K1H 8M5 Ontario, Canada; 3 Centre for Neural Dynamics, University of Ottawa, Ottawa, K1N 6N5 Ontario, Canada

## 

When processing sensory input, it is of vital importance for the neural systems to be able to discriminate a novel stimulus from the background of redundant, unimportant signals. Neural mechanisms responsible for prediction and cancellation of redundant information could be an efficient way to achieve such discrimination. While the concrete mechanisms that the brain employs for this task are presently unknown, a network able to perform this cancellation is thought to exist in the electrosensory lateral line lobe (ELL) of weakly electric fish [1]. This fish emits a high-frequency (600-1000 Hz) sinusoidal electric organ discharge (EOD) into its environment to sense its surroundings and communicate to conspecifics. Small objects such as prey create spatially localized amplitude modulations (AMs) of the EOD, whereas tail bending or communication signals induce spatially global AMs [2]. These AMs are detected by electroreceptors that densely cover the body of the fish, and provide feedforward input to pyramidal cells in the ELL. It is known that a subpopulation of such pyramidal cells, the superficial pyramidal (SP) cells, remove low-frequency predictable global signals (i.e. tail bending) from their input to maximize detection of novel local stimuli (i.e. prey) [1]. This is presumably achieved using a feedback pathway involving the granule cell layer (a cerebellar-like structure known as EGp). These granule cells connect to SP cells via parallel fibers (PFs) which may be acting as delay lines segregated into frequency channels to destructively interfere with the global stimulus. Recent *in vitro* studies found a novel burst timing-dependent learning rule which would be able to shape this feedback [3].

Following a previous work [4], we study the cancellation of low-frequency simple redundant signals, i.e. sine waves, in the ELL of the weakly electric fish. The study combines *in vitro* data, *in vivo* electrophysiology recordings from neurons in the ELL and numerical modeling to address this issue. More precisely, we model the neural network responsible for signal cancellation in the ELL of the fish, and compare our predictions with electrophysiology data recorded *in vivo *[4]. In the model, we assume the presence of: 1) stimulus-driven feedback to the SP neurons, 2) a large variety of temporal delays in the PFs transmitting such feedback, and 3) burst-induced long-term plasticity. We show that the modeled network is able to efficiently cancel global redundant signals by shaping the feedback as a negative image of the global signal arriving to the SP cells. Such negative image is generated via the burst-induced anti-Hebbian learning rule in the PF-SP cell synapses, while the full period of the signal is covered by the incoming feedback due to the wide range of PF delays present in the network. The cancellation is found to be in agreement with *in vivo* recordings, and it is strong for signals with frequencies up to 16 Hz, enabling a clearer background above which to detect relevant non-repetitive stimuli such as prey signals (and thus to better capture the prey). Due to the importance of the phase-relationship between the feedback and the stimulus, the mechanism is found to be frequency-specific, suggesting the presence of multiple frequency channels as observed *in vivo *[4]. Interestingly, our model predicts that the cancellation is maintained for signals with different AM strengths (i.e. contrasts). Such contrast-invariance is highly desirable since natural signals would display different contrasts depending, for instance, on the distance between the fish and the origin of the EOD perturbation.
